# The brief international cognitive assessment for multiple sclerosis (BICAMS): normative values with gender, age and education corrections in the Italian population

**DOI:** 10.1186/s12883-014-0171-6

**Published:** 2014-09-10

**Authors:** Benedetta Goretti, Claudia Niccolai, Bahia Hakiki, Andrea Sturchio, Monica Falautano, Eleonora Minacapelli, Vittorio Martinelli, Chiara Incerti, Ugo Nocentini, Monica Murgia, Giuseppe Fenu, Eleonora Cocco, Maria Giovanna Marrosu, Elisabetta Garofalo, Ferdinando Ivano Ambra, Maurizio Maddestra, Marilena Consalvo, Rosa Gemma Viterbo, Maria Trojano, Nunzia Alessandra Losignore, Giovanni Bosco Zimatore, Erika Pietrolongo, Alessandra Lugaresi, Dawn Langdon, Emilio Portaccio, Maria Pia Amato

**Affiliations:** Department of NEUROFARBA, Section Neurosciences, University of Florence, Florence, Italy; Don Gnocchi Foundation, Florence, Italy; Neurological Department, IRCCS San Raffaele, Milan, Italy; Department of Clinical and Behavioural Neurology, Santa Lucia Foundation, Rome, Italy; Department of Public Health and Clinical and Molecular Medicine, Multiple Sclerosis Center, University of Cagliari, Cagliari, Italy; Ospedale dei Colli Monaldi Cotugno CTO, UOC Neurologia STROKE UNIT, University of Napoli, Napoli, Italy; U.O. Neurologia, Hospital of Lanciano, Chieti, Italy; Department of Basic Medical Sciences, Neurosciences and Sense Organs, University of Bari Aldo Moro, Bari, Italy; U.O.C. Neurologia P.O. Dimiccoli, Barletta, Italy; Department of Neuroscience and Imaging, University “G. d’Annunzio” of Chieti-Pescara, Chieti, Italy; Royal Holloway, University of London, Surrey, UK

**Keywords:** Multiple sclerosis, Cognitive impairment, Assessment tool, Italian normative values

## Abstract

**Background:**

BICAMS (Brief International Cognitive Assessment for Multiple Sclerosis) has been recently developed as brief, practical and universal assessment tool for cognitive impairment in MS subjects. It includes the Symbol Digit Modalities Test (SDMT), the California Verbal Learning Test-2 (CVLT2) and the Brief Visuospatial Memory Test–Revised (BVMT-R) . In this study we aimed at gathering regression based normative data for the BICAMS battery in the Italian population.

**Methods:**

Healthy subjects were consecutively recruited among patient friends and relatives. Corrections for demographics were calculated using multivariable linear regression models. Test-retest reliability was assessed using the Pearson correlation coefficient.

**Results:**

The BICAMS battery was administered to 273 healthy subjects (180 women, mean age 38.9 ± 13.0 years, mean education 14.9 ± 3.0 years). Test-retest reliability was good for all the tests.

**Conclusions:**

The study provided normative data of the BICAMS for the Italian population confirming good test-retest reliability which can facilitate the use of the battery in clinical practice, also for longitudinal patient assessments.

## Background

Cognitive impairment (CI) affects up to 65% of multiple sclerosis (MS) subjects, involves all the disease subtypes and can be documented from the very beginning of the disease [1,2]. Moreover, once established, it tends to progress over time, sometimes independently of the accumulation of physical disability [1]. Beyond the effects of physical disability, CI has an important negative impact on patient performance in everyday activities and is the main factor leading to reduced employment or unemployment [1] in this population of young adults. CI also affects social activity, general independence, coping, rehabilitation progress, treatment adherence and mental health of MS patients [3]. The neuropsychological profile typically involves impaired complex attention and information processing speed, episodic memory and executive functions, whereas language and general intelligence are usually spared [1].

For this purpose, over the last decades, assessment of MS-related CI has received growing attention and different neuropsychological batteries have been proposed. The most commonly utilized batteries are the Brief Repeatable Battery of Neuropsychological tests (BRB-N) [4] and the Minimal Assessment of Cognitive Function in MS (MACFIMS) [5]. These batteries were recognized as highly accurate in MS patients, however, their implementation in clinical practice is limited by their time-consuming nature (respectively 45 and 90 minutes) and the need of supervision and interpretation by experienced neuropsychologists. Therefore, there has been considerable effort over the past decade to streamline the neuropsychological assessment in MS, by developing brief assessment tools that can be incorporated in everyday patient assessment. In particular, recently, a Brief International Cognitive Assessment for MS (BICAMS) [3,6] is recommended as an international, validated and standardized brief cognitive test. This battery takes about 15 minutes for administration and can be used in everyday clinical practice, even in small MS centers with few staff members who may not have neuropsychological training [3,6]. It could also be integrated into more detailed cognitive assessments and used as a brief assessment tool to identify patients who require a more comprehensive evaluation by specialized personnel. The objective of the this Italian multicentric collaborative study was to provide BICAMS norms in the Italian population, with corrections for gender, age and educational level, as well as to assess test-retest reliability in our population, following recommended international standards for validation [6].

## Methods

### Subjects

A total of 273 healthy subjects (180 women; 93 men) were recruited from the community in ten Italian sites representative of the whole national territory (Bari, Barletta, Cagliari, Catania, Chieti, Florence, Lanciano, Milan, Naples and Rome). Exclusion criteria were neurological and major psychiatric illness, history of learning disability, serious head trauma (causing coma and/or neuropsychological dysfunctions), alcohol or drug abuse as well as major medical illness. All subjects had adequate vision and hearing to undergo the tests. Two hundred and forty-three healthy controls agreed to undergo a second assessment using the same version of the test, three weeks apart. All the participants in the study provided their informed consent and the study was approved by the ethic committee of the University of Florence.

### Neuropsychological test procedures

In each site, the subjects were examined by the same neuropsychologist who had participated in a common training session, in order to ensure uniform administration, data recording and scoring procedures.

Tests were administered in a standardized manner, during daytime, in a quiet room, and in a fixed order: SDMT [4], CVLT2 first five trials [7] and BVMT-R first three recall trials [8]. Administration of the whole battery took 13.7 ± 3.3 minutes (median value 15 minutes).

- SDMT presented a series of nine symbols, each paired with a single digit in a key at the top of an 8.5 -1-inch sheet. The remainder of the page presented a pseudo-randomized sequence of symbols. Participants responded by voicing the digit associated with each symbol as quickly as possible. The SDMT required both rapid information processing and visual scanning, and to a lesser extent, working memory. The dependent variable was the total number of correct responses in 90 seconds.
- CVLT-II is a measure of verbal learning and memory. The ability to learn a 16-word list (List A) was first examined over the course of five trials. Examiner read 16 words and asked participants to repeat as many words as possible. The entire List A was repeated each time. The dependent variable we considered was the total number of words recalled over five learning trials (Total Learning, TL).
- BVMT-R is a measure of visuospatial learning and memory. These were tested by exposing of the participants to a matrix of six simple abstract designs for 10 seconds followed by an unaided recall. Participants were asked to reproduce the designs using paper and pencil, taking as much time as needed for reproduction. Each design received a score 0, 1, or 2 based on accuracy and location scoring criteria. The dependent variable was total recall score across the three trials.

This is the first implementation of the BICAMS in Italy; therefore the battery was translated and culturally adapted for the Italian population. In particular, for visual stimuli, the presence of any semantic associations to stimuli in Italian culture and language was screened. Moreover, the CVLT-II was forward translated from English into Italian by a professional native-speaking Italian translator, matching new words on word frequency and appropriate similarity of meaning.

### Statistical analysis

Group comparisons were assessed through the Student’s t –test for unpaired samples, the Mann–Whitney test and the χ2 test, when appropriate. Regression-based norms were calculated following the previously described procedure applied for the MACFIMS [9]. In particular, the control group’s raw scores on each neuropsychological measure were converted to scaled scores (*M* = 10, *SD* = 3) using the cumulative frequency distribution of each measure. We then regressed the resulting scaled scores on age, age-squared, gender (male = 1; female = 2), and education, entered en bloc. The inclusion of a term of age-squared allowed us to consider the nonlinear relationship between age and cognition. The assumptions of regression analysis were tested by conducting a Kolmogorov-Smirnov test to evaluate the normality of the residuals. The Kolmogorov-Smirnov test should not be significant. Moreover, significant demographic predictors of scaled scores were assessed through backward stepwise multivariable regression models.

The normative data can be established as it follows: the participants’ raw test scores must be converted to scaled scores using the raw-to-scale-score conversions derived from the healthy controls. Next, the multiple regression equations derived from the healthy controls must be applied to compute demographically predicted scores for each participant. These predicted scores must be then subtracted from each participant’s actual scores and the differences divided by the standard deviation of the controls group’s raw residuals for each measure. Finally, the resulting values can be converted to *T* scores and performance on each neuropsychological measure classified as either intact ( *T* > 35) or impaired ( *T* ≤ 35).

Test-retest reliability was assessed using the Pearson correlation coefficient. We used alpha = 0.05 as a significance level for including the regression coefficient in the formula. All analyses were performed using the SPSS 20 running on Windows (SPSS, Chicago, IL, USA).

## Results

Tables 1 and 2 show the main characteristics of the subjects who completed the test battery at baseline and re-test assessments. Distribution of different classes of ages and education is reported in Table 2. Classes of age were evenly distributed; more educated subjects were predominantly represented (54.6%). Table 1 lists mean baseline and re-test scores of the BICAMS components. In the 243 healthy subjects who were re-assessed after three weeks, using the same versions of the tests, mean scores were significantly higher on all the cognitive measures (p < 0.0001).

**Table 1:** **Characteristics of the study sample**

#	**273** **(baseline sample)**	**243** **(re**-**test sample)**		**p**
		**Baseline**	**Re**-**test**	
Females/Males	180/93	158/85		n.s.
Mean age: years ± SD (range) Median	38.9 ± 13.0 (18–65); 38.1	38.2 ± 13.1 (18–65); 35.3		n.s.
Mean education: years ± SD (range) Median	14.9 ± 3.1 (5–21); 16.0	14.9 ± 3.0 (5–21); 16.0		n.s.
SDMT	56.3 + 11.3 (30–91)	56.2 ± 11.6 (30–91)	60.3 + 12.0 (29–107)	< 0.001
CVLT-II	56.3 + 9.0 (31–78)	56.2 ± 9.1 (31–78)	64.2 + 10.1 (34–80)	< 0.001
BVMT-R	27.9 + 6.1 (11–36)	28.1 ± 6.2 (11–36)	31.3 + 5.5 (12–36)	< 0.001

*SD*; standard deviation; *ns*: not significant; *SDMT*; Symbol Digit Modalities Test; *CVLT*-II; California Verbal Learning Test second edition; *BVMT*-*R*; Brief Visuospatial Memory Test Revised.

**Table 2:** **Classes of age and education**

**Classes of age** **(** **Years** **)**	**# (%)**	**Gender**	**Education**	
			≤**13**	>**13**
18-30	96 (35.1)	Males #32	14	18
		Females#64	21	43
31-40	51 (18.7)	Males #20	8	12
		Females#31	12	19
41-50	60 (22)	Males #15	8	7
		Females#45	24	21
51-65	66 (24.2)	Males #26	16	10
		Females#40	21	19
**Classes of education** **(** **Years** **)**	# (%)	**Gender**	**Age years** ± **SD**	
0-5	3 (1.1)	Males #0	53.4 ± 3.9	
		Females#3		
6-8	15 (5.5)	Males #10	47.2 ± 14.8	
		Females#5	42.5 ± 3.0	
9-13	106 (38.8)	Males #36	39.6 ± 14.0	
		Females#70	40.2 ± 13.6	
>13	149 (54.6)	Males #47	37.6 ± 12.6	
		Females#102	37.0 + 12.2	

*M*/*F*; male/female; *SD*; standard deviation.

Table 3 reports the raw to scaled scores (*M* = 10, *SD* = 3) conversion using the cumulative frequency distribution of each measure of the BICAMS. Table 4 shows the normal control regression models for the BICAMS. All models include age, age-squared, sex (male = 1; female = 2), and education. The Kolmogorov-Smirnov test on the distribution of the residuals was negative for all the three tests (p > 0.06).

**Table 3:** **Raw score to scaled score conversions** (**baseline sample**)

	**Raw scores**		
**Scaled score**	**SDMT** **(#** **273** **)**	**CVLT**-**II** **(#** **273** **)**	**BVMRT** **(#** **273** **)**
18	>87	>74	36
17	84-87		
16	79-83	72-74	
15	73-78	70-71	35
14	70-72	66-69	34
13	65-69	63-65	33
12	62-64	61-62	32
11	58-61	58-60	30-31
10	53-57	55-57	28-29
9	50-52	52-54	25-27
8	47-49	48-51	23-24
7	44-46	45-47	20-22
6	39-43	42-44	17-19
5	37-38	38-41	14-16
4	34-36	35-37	12-13
3	32-33		11
2	<32	<35	<11

*BICAMS*; Brief International Cognitive Assessment for Multiple Sclerosis; *SDMT*; Symbol Digit Modalities Test; *CVLT*-II; California Verbal Learning Test second edition; *BVMT*-*R*; Brief Visuospatial Memory Test Revised.

**Table 4:** **Final regression models for BICAMS measures**

**Measure**	**Predictor**	**B**	**Standard error B**	**T**	**Standardized B**	**Total R square**
**SDMT**	(constant)	10.854	1.960	5.538		
	Age	0.040	0.092	0.436	0.177	
	Age^2^	−0.002	0.001	−1.472	−0.598	
	Gender	−0.192	0.344	−0.559	−0.031	
	Education	0.060	0.055	1.103	0.062	0.194
**CVLT**-**II**	(constant)	4.989	2.096	2.381		
	Age	0.118	0.099	1.197	0.509	
	Age2	−0.002	0.001	−1.635	−0.696	
	Gender	0.823	0.367	2.242	0.130	
	Education	0.178	0.058	3.045	0.180	0.113
**BVMRT**	(constant)	12.694	2.310	5.496		
	Age	−0.039	0.109	−0.361	−0.151	
	Age2	−0.001	0.001	−0.459	−0.192	
	Gender	−0.671	0.405	−1.659	−0.094	
	Education	0.101	0.064	1.567	0.091	0.144

*BICAMS*; Brief International Cognitive Assessment for Multiple Sclerosis; *SDMT*; Symbol Digit Modalities Test; *CVLT*-II; California Verbal Learning Test second edition; *BVMT*-*R*; Brief Visuospatial Memory Test Revised.

These models can be used to convert MS raw scores to regression-based T scores. For example, consider a 38-year-old female MS patients with 18 years of education. Her predicted scaled score on the CVLT-II is 11.435 [4.989 + 38(0.118) + 38^2^(−0.002) + 2(0.823) + 18(0.178)]. Her actual CVLT-II score of 53 corresponds to a scaled score of 9, according to Table 3. We then divide the difference between her actual and predicted scaled score (9–11.435 = −2.453) by the standard deviation of the residual reported in Table 5 (2.842), obtaining a z score of −0.86, which equals a T score of 41.4.

**Table 5:** **Standard deviation of the residual from healthy controls**

	**SD residual**
**SDMT**	2.658
**CVLT**-**II**	2.842
**BVMRT**	3.133

*SD*: Standard Deviation; *SDMT*; Symbol Digit Modalities Test; *CVLT*-II; California Verbal Learning Test second edition; *BVMT*-*R*; Brief Visuospatial Memory Test Revised.

In the backward stepwise multivariable regression models, higher age was associated with a worse performance on the three tests (SDMT: age-squared B = −0.001, p < 0.001; CVLT-II: age-squared B = −0.001, p = 0.001; BVMRT: age-squared B = −0.001, p < 0.001). The CVLT-II was also influenced by education (B = 0.181, p = 0.002), with higher education associated with better performance, and by gender (B = 0.847, p = 0.022), with women performing better than men.

Finally, test-retest reliability for raw scores on each test was good for all the three tests (Table 6).

**Table 6:** **Test**-**retest reliability of the BICAMS tests**

**Test**	**r**	**p**
CVLT-II	0,763	< 0.001
SDMT	0,815	< 0.001
BVMT-R	0,744	< 0.001

*BICAMS*; Brief International Cognitive Assessment for Multiple Sclerosis; *SDMT*; Symbol Digit Modalities Test; *CVLT*-II; California Verbal Learning Test second edition; *BVMT*-*R*; Brief Visuospatial Memory Test Revised.

## Discussion

The BICAMS [3] is a well-validated, rapid and reliable instrument, which is optimized also for small MS centres and international use. This brief cognitive tool should not replace more comprehensive evaluation of the cognitive function, such as MACFIMS [5] or BRB-N [4]; instead, it will increase accessibility for cognitive assessment in non-specialized centres and it can also be integrated into more detailed cognitive assessments and used as a brief assessment tool in all MS centres. Translation and validation of the BICAMS is ongoing in several countries. In a recent study, BICAMS has been validated in the Czech population. This study showed that BICAMS accuracy and reliability are similar to those of the MACFIMS, so that it can be recommended for further clinical use [10]. These BICAMS psychometric characteristics have been confirmed in an Iranian sample [11].

In the present study, we obtained normative values for the BICAMS tests in a large Italian population representative of the whole national territory, providing corrections for demographics. Our raw scores, compared with results in the Czech sample, showed lower means for the SDMT and CVLT-II, whereas the scores of BVMT-R were almost overlapping. The Czech sample was younger than the currently described Italian sample (mean age 34.0 years versus 38.9 years, respectively). However, scores on these tests were also higher for an American sample with a similar age range, which highlights the need for appropriately validated national samples to take account of translation, cultural and educational differences [6]. These results highlight the subtle linguistic and cultural differences that occur when tests are translated and the importance of national validation data.

Comparing our current data for the SDMT with Italian normative data for the BRB [12] we found that mean scores of SDMT in the present study were higher (56.3 vs 50.9). This may be due to younger age of the subjects included in the present study (p < 0.05).

It is noteworthy that our study confirmed good test-retest reliability of the BICAMS tests which is an essential requirement in longitudinal assessments of the patients. In this respect, test-retest reliability was higher (r = 0.815) for the SDMT, which is in line with previous reports [13]. It has to be noted that high reliability would be likely due to practice effects with the same form tested. This finding should be confirmed using alternate forms of the tests. [14].

Our sample tended to include mostly young and middle-aged subjects with higher educational levels. Therefore, the demographic corrections may be not be precise for older subjects with less education; however this group tend to be less represented in an MS clinical population.

## Conclusions

In conclusion, our results provide BICAMS norms for use with an Italian population and provide evidence for the robust psychometric properties of the BICAMS scales in a fourth national population. The battery was completed with a median time of 15 minutes, confirming that it is a brief tool appropriate for a broad range of clinical and research uses. We have also reported the largest test-retest sample data for BICAMS, which affirms its suitability for longitudinal assessment. The next step in the validation process will be the comparison of results obtained in our healthy population with those of an Italian MS sample, in order to evaluate the criterion-related validity of the BICAMS.

## Abbreviations

MS: Multiple sclerosis
BICAMS: Brief International Cognitive Assessment for Multiple Sclerosis
SDMT: Symbol Digit Modalities Test
CVLT-II: California Verbal Learning Test – II
BVMT-R: Brief Visuo-spatial Memory Test – Revised
CI: Cognitive impairment
BRB-N: Brief Repeatable Battery of Neuropsychological tests
MACFIMS: Minimal Assessment of Cognitive Function in Multiple Sclerosis
TL: Total learning
